# Dr. Trotter's New Work, on Indigestion

**Published:** 1806-03

**Authors:** T. Trotter

**Affiliations:** Newcastle


### *To the Editors of the Medical and Physical Journal.*

GENTLEMEN,

YOU were kind enough, a few months ago, to announce my intended Publication, "*On the increasing prevalence, prevention and treatment of those diseases, commonly called nervous bilious indigestion, &c.* It however happened, as I proceeded in my inquiry, that the importance of the subject very much rose in my opinion, and which has retarded the work from being put to the press Another cause of delay has been, the sending of queries to several physicians, who had long practiced in the East and West Indies. Answers to these being mostly received, the book will now be forth-coming in due time.

Although

Although much has of late been written on these complaints, I do not find myself anticipated in the inquiry; or the ground that I have taken in the investigation peculiarly occupied. Dr. Cheyne's work, called the "*English Malady*," though, in many respects, a book of learning and genius, is obsolete in both doctrine and practice. The more scientific labors of Whytt, intitled "*Observations, &c.*" must always be referred to with advantage by the practical physician: I have therefore encroached but little on his method; and have chiefly directed myself to an analysis of the manners and customs of modern society, as they influence health, and give a peculiar character to some diseased movements in both body and mind. Few medical philosophers, who appreciate the pleasure of existence by the full enjoyment of the rational faculty with corporealaction, will contend that this class of diseases is unworthy of serious contemplation. In the present day, they form by far the largest proportion of the whole which claim professional aid. Since May 1802, of three hundred and eighty-four poor persons in this town and neighbourhood, who have applied to me for advice, three hundred have complained in some shade or gradation of these ailments. So large a number, affords to a reflecting mind, a melancholy picture of the condition of society. Many of them having occasional retoritial symptoms, had been most wantonly treated by *calomel;* and the dyspeptic symptoms much aggravated thereby. When these disorders arrive at the extent they have done at this time, they indicate a degree of dotage and decline in the intellectual and bodily powers of the human race. They are the offspring of an age, wallowing in wealth, indolence, and luxury. And as we view them with a philosophical eye, we perceive something like a physical necessity for the regeneration of mankind. But I must stop here, and give you a kind of syllabus of my work:

INTRODUCTION.

CHAP. I. The health of the savage state, compared with modern times.

CHAP. II. The medical description of the inhabitants of a city or town; or, analysis of modern society, viz.

1. Literary men.
2. Men of business.
3. The idle and dissipated.
4. The artificer and manufacturer.
5. Those employed in drudgery.
6. Persons returned from the East and West Indies.

7. The

7. The female sex; consisting of the higher, midling, and lower orders of women.

CHAP. III. The remote causes of nervous diseases, &c. viz. in,
1. Air;
2. Exercise;
3. Food;
4. Clothing;
5. Passions of the mind;
6. Intense study;
7. Lactation;
8. Sexual intercourse;
9. Climate;
10. Medicine.

CHAP. IV. Influence of these diseases, on the character of nations and domestic happiness.

*a.* Rise and decay of a commercial town, an epitome of a state.
*b.* Diseases attending wealth.
*c.* Barbarian and commercial Britain compared.
*d.* The dread of French invasion, compared with that of Julius Cæsar.
*e.* The Mortello Tower, and inundation of Essex, contrasted with the walls of Adrian and Severus.
*f.* Other nervous symptoms of a nation; hoarding up the gold coin, &c
*g.* Board of agriculture, a step to regenerating society. Another Addison wanted to regenerate morals.
*h.* Bilious temperament of Buonoparte.
*i.* Character of nervous persons, effects on private life.
*k.* The greatest vices and virtues found in the nervous temperament, &c. &c.

CHAP. V. History and progress of these diseases.
Predisposition or nervous temperament.
———— hereditary.
———— acquired.
———— gouty.
Symptoms of nervous and bilious affection, &c.

CHAP. VI. General doctrine of these diseases.

CHAP. VII. Prevention and treatment.

Should this plan suggest any thing to your numerous readers, I shall be much obliged by their communications. I observe with pleasure a strong coincidence of opinion on this this subject, between your lively correspondent Dr. Reid and myself.

---

In consequence of *peculiar occurrences*, in this vicinity, I beg leave to withdraw my promise of sending to your Journal, the remainder of the case, *gastrodynia*, in your 68th No. The reflections upon it being allied to my present labors, will have a place in this Volume. You will recollect, I was charged with *presumption* in offering sentiments on a disease, opposite to those of Dr. Clark and Mr. Ingham. The mode of attack against me has, however, been varied; I must therefore alter my means of defence. Had I returned to the neighbourhood, where I received the first rudiments of my medical studies, to insult it by a fortune acquired by gross peculation in the service of my country; or to dazzle the world by a splendid equipage, purchased by the rapine of plundered provinces; I might have been an object for censure and malignity: but at a moment when I stood between the *sick bed* of an amiable woman and the *grave*, in opposing an instance of the greatest violation of medical practice that is to be found in the records of Physic, it is strange indeed.— Every independent mind will view the detail of these circumstances with disgust; for the same attempts which have been made to take the *bread from my mouth*, may be employed against every professional man, who weilds the duties of science and candor, against ignorance. *Ruat cœlum fiat Justitia.*

I observe your learned correspondent, Dr. Thomas, in your last number, takes notice of Mr. Fogo's remarks on the case of *gastrodynia*. I did not reply to Mr. F. particularly, because I thought he drew conclusions that were not to be justified from the premises. But if the attending physician and apothecary, could call the spasmodic affections of a nervous temperament, a *pleurisy*, Mr. Fogo might very well call them *hepatitis*.

I am happy to inform you, that contrary to all expectation, my case of "*Mollities ossium*," in your 75th No. is completely recovered. The young gentleman, after some interval, returned to the use of his medicine with increased advantage, till all unpleasant symptoms disappeared. Every palsied weakness is now restored to strength; and not even any deformity of the cervical vertebra, that was partially displaced, is to be observed.

I am, &c.

T. TROTTER, M. D.

