# Errata

**Published:** 1806-03

**Authors:** 


215, l. 4, from the bottom (in those copies where not corrected) for *preservation*, read *prevention*.

